# Headaches in Pediatric Patients during the Past Decade: Comparative Analysis by Age Group from a Multicenter Study in Korea

**DOI:** 10.3390/brainsci14100951

**Published:** 2024-09-24

**Authors:** Eu Gene Park, Seung Beom Han, Jin Lee, Jee Min Kim, Ji Yoon Han

**Affiliations:** 1 Department of Pediatrics, College of Medicine, The Catholic University of Korea, Seoul 06591, Republic of Korea; eugene.park@catholic.ac.kr (E.G.P.); beomsid@catholic.ac.kr (S.B.H.); pedleejin@naver.com (J.L.); 2 Department of Pediatrics, Incheon St. Mary’s Hospital, College of Medicine, The Catholic University of Korea, Seoul 21431, Republic of Korea; 3 Department of Pediatrics, Seoul National University Hospital, Seoul 03080, Republic of Korea; jee.raphaela.kim@gmail.com; 4 Department of Pediatrics, Daejeon St. Mary’s Hospital, College of Medicine, The Catholic University of Korea, Seoul 34943, Republic of Korea

**Keywords:** headache, children, adolescents

## Abstract

Background: Headache is a common complaint during childhood and adolescence. It is important to be aware of the characteristics of pediatric headaches in order to make a precise and timely diagnosis. This study investigated the clinical characteristics of pediatric headaches according to the underlying etiology and age group. Methods: We retrospectively reviewed the medical records of 3374 pediatric patients (2667 with primary headache [PH] and 707 with secondary headache [SH]) who presented with headaches at two centers between January 2012 and November 2023. Results: The incidence of PH was significantly higher in adolescents (40.1% vs. 22.9%), whereas that of SH was considerably higher in preschoolers (37.5% vs. 16.3%) (*p* < 0.001). The prevalence of headaches attributed to infection was significantly higher in preschool and school-aged children (90.8% vs. 80.2%, *p* < 0.001); adolescents exhibited significantly higher frequencies of cranial and cervical vascular disorders (3.7% vs. 1.3%, *p* = 0.044) and psychiatric disorders (5.6% vs. 0.6%, *p* < 0.001). Statistically significant differences were observed between preschool/school-aged children and adolescents in terms of headache characteristics and the prevalence of headache-associated symptoms (60.4% vs. 74.1%, *p* < 0.001 in PH), neurologic abnormalities (10.2% vs. 23.6%, *p* < 0.001 in PH; 2.4% vs. 11.7%, *p* < 0.001 in SH), and headache triggers (19.9% vs. 24.2%, *p* = 0.008 in PH; 2.0% vs. 8.0%, *p* < 0.001 in SH). Conclusions: Recognizing the etiology and age-specific differences in the clinical characteristics of headaches is crucial for an accurate and timely diagnosis. Tailoring the diagnostic approach accordingly helps to achieve optimal outcomes in pediatric patients with headaches.

## 1. Introduction

Headache is a common complaint among children and adolescents, leading to referrals to pediatric neurologists. Population-based studies estimated that the global prevalence of headaches in this age group is 58.4% [1]. While headaches are uncommon before the age of 4, their prevalence increases throughout childhood, peaking around 13 years of age in both sexes [2]. Furthermore, 3.6% of 8-year-olds and 10.7% of 15-year-olds experience at least one headache weekly [3]. A Finnish study investigated over 1000, 7-year-old children and found that the prevalence of migraine increased from 14.5 per 1000 in 1974 to 91.9 per 1000 in 2002 [4]. Additionally, a study in Norway demonstrated that the overall prevalence of recurrent headaches among adolescents increased from 30.3% in 1995–1997 to 35.4% in 1999–2001 [5]. Most pediatric headaches can be classified as either primary headaches, such as migraines and tension-type headaches, or secondary headaches due to non-life-threatening conditions, such as upper respiratory infections, sinusitis, or mild head trauma [6]. The incidence of serious intracranial diseases requiring urgent identification in pediatric headaches is low, ranging from 0.5% to 1.0% [7,8,9].

Primary headache disorders are the leading cause of neurological disabilities in children and adolescents [10]. The impact of headaches on the quality of life is substantial, limiting social activities, physical activity, and school attendance, which contribute to poorer learning outcomes and an increased risk of dropping out of school [11,12]. Moreover, children and adolescents with migraine tend to experience more depressive and anxiety symptoms and have higher odds of developing depressive and anxiety disorders than their peers without migraine [13]. Hence, early diagnosis and treatment are crucial for enhancing short-term outcomes related to headaches and improving long-term results [14].

Most children do not present with classic symptoms of primary headache that fit the diagnostic criteria [15]. Moreover, their ability to describe headaches is limited by their developmental stage [16]. Therefore, establishing an accurate diagnosis of headaches in pediatric patients is challenging. Uncertainty in the diagnostic process may lead to superfluous testing and unnecessary concerns for the patients and their parents. Hence, clinicians must be aware of the characteristics of pediatric headaches to make an early diagnosis and ensure optimal patient outcomes. However, there are limited data on the clinical characteristics of pediatric patients with headaches across different ages, including preschool period, childhood, and adolescence.

This study aimed to investigate the clinical characteristics of pediatric headaches according to the underlying etiology and age group to enhance the understanding of pediatric headaches for improved clinical practice.

## 2. Materials and Methods

We performed a retrospective medical chart review of pediatric patients who presented with headaches at Incheon St. Mary’s Hospital, Incheon, Korea, and Daejeon St. Mary’s Hospital, Daejeon, Korea. The patients were recruited between January 2012 and November 2023. We excluded patients with known systemic diseases, intracranial lesions, or a history of head trauma within the preceding three months.

The data obtained included age, sex, location, characteristics, intensity, frequency, duration of headache, associated symptoms, triggering factors, analgesic usage, preexisting illnesses, family history of migraine, and neuroimaging findings. Pain intensity was measured using the visual analog scale. All neuroimaging findings were interpreted by neuroradiologists at each institution.

Headaches were classified according to the International Classification of Headache Disorders, third edition, proposed by the Committee of the International Headache Society [17]. A headache that did not clearly match a defined type was included as an unclassified headache. The diagnosis of a headache attributed to a psychiatric disorder was based on a direct temporal relationship between the psychiatric disorder and the onset or worsening of the headache, as well as when the symptoms could not be fully explained by a primary headache disorder alone. According to the underlying cause of the headache, the patients were divided into the primary headache group and the secondary headache group. To evaluate the clinical characteristics of headaches at various ages, we classified the patients into two groups: those aged ≤12 years (preschool and school-aged children) and those aged 13–18 years (adolescents).

All statistical analyses were performed using SPSS software (version 21.0; IBM Corp., Armonk, NY, USA). Numerical data, including age, were compared using Student’s *t*-test or the Mann–Whitney U test. Categorical data, such as the demographic and clinical characteristics of headaches, were analyzed using Fisher’s exact test or the chi-square test. Fisher’s exact test was used when more than 20% of the cells had expected frequencies of <5. Statistical significance was set at *p* < 0.05.

This retrospective study was approved by the Institutional Review Board of the Catholic Medical Center (IRB OC23RASI0159, DC24RASI0047). The need for informed consent was waived due to the retrospective nature of this study.

## 3. Results

### 3.1. Patient Characteristics

A total of 3374 patients (1593 males and 1781 females) who presented to the hospital with headaches were identified between January 2012 and November 2023. The median age at the initial visit was 11 years (interquartile range (IQR), 7–14 years). Of the 3374 patients, 2667 (79.0%) presented with primary headache, and 707 (21.0%) presented with secondary headache. The most common primary diagnosis was migraine (n = 1140, 42.7%; 993 cases of migraine without aura and 147 cases of migraine with aura), followed by unclassified headache (n = 1053, 39.5%) and tension-type headache (n = 474, 17.8%). The most common secondary diagnosis was headache attributed to infection (n = 625, 88.4%), followed by headache attributed to disorder of the cranium, neck, eyes, ears, nose, sinuses, teeth, mouth, or other facial or cervical structure (n = 39, 5.5%), headache attributed to cranial and cervical vascular disorder (n = 13, 1.8%), headache attributed to non-vascular intracranial disorder (n = 13, 1.8%), headache attributed to psychiatric disorder (n = 12, 1.7%), and headache attributed to disorder of homeostasis (n = 5, 0.7%) (Table 1).

The demographic and clinical characteristics of the primary and secondary headache groups are shown in Table 2. The proportion of primary headaches was significantly higher in adolescents (40.1% vs. 22.9%), whereas the proportion of secondary headaches was considerably higher in preschoolers (37.5% vs. 16.3%, *p* < 0.001). A significantly higher number of male patients were diagnosed with secondary headaches (54.3% vs. 45.3%) (*p* < 0.001). Statistically significant between-group differences were observed in headache characteristics, including localization, character, severity, duration, and clinical course. The primary headache group experienced more recurrent or chronic pain lasting less than an hour, which was localized, throbbing, prickling, or pressure-like, and they often reported daily headaches compared to the secondary headache group. In contrast, the secondary headache group presented with more acute pain, characterized by a combination of diffuse and localized pain, lasting more than an hour and exhibiting varying patterns with mixed characteristics. Both groups experienced moderately severe pain. However, in the primary headache group, the proportion of patients reporting mild pain (20.5% vs. 10.2%) or severe pain (18.1% vs. 8.2%) was significantly higher than that in the secondary headache group (*p* < 0.001). The primary headache group had a significantly higher proportion of individuals with neurological abnormalities (15.6% vs. 4.5%) and identifiable triggers (21.6% vs. 3.4%) than the secondary headache group (*p* < 0.001). Additionally, the secondary headache group had a significantly higher proportion of patients with headache-associated symptoms than the primary headache group (74.0% vs. 65.9%) (*p* < 0.001). Patients with primary headaches exhibited a significantly greater likelihood of experiencing headaches that awaken them from sleep (18.9% vs. 9.3%) and headaches that occurred on or soon after waking up in the morning (27.6% vs. 8.2%) than those with secondary headaches (*p* < 0.001). The proportion of patients with a family history of migraine was considerably higher in the primary headache group than in the secondary headache group (31.2% vs. 4.5%, *p* < 0.001).

### 3.2. Comparison of Primary Headache Characteristics by Age Groups

Of the 2667 patients, 1597 (59.9%) were preschool and school-aged children, and 1070 (40.1%) were adolescents. As shown in Table 3, the proportion of male patients was significantly higher among preschool and school-aged children (50.8% vs. 37.1%, *p* < 0.001). More than half of the patients had a normal body mass index (BMI) in each age group. In particular, preschool and school-aged children were more likely to have a BMI below the normal range (38.1% vs. 15.5%, *p* < 0.001), whereas adolescents were more frequently above the normal BMI range (19.4% vs. 9.0%, *p* < 0.001). The most common diagnoses were unclassified primary headaches in preschool and school-aged children (48.3%) and migraines with or without aura in adolescents (56.9%). No significant difference in the proportion of patients with tension-type headaches was observed between the two groups (18.4% vs. 16.8%, *p* = 0.293). Preschool and school-aged children often experienced acute headaches characterized by a combination of diffuse and localized pain lasting less than 30 min; however, adolescents more frequently experienced recurrent and chronic headaches with diffuse pain lasting longer than an hour. Both groups experienced moderately severe pain. However, in preschool and school-aged children, the proportion of patients reporting mild pain was significantly higher than that in adolescents (26.0% vs. 12.3%, *p* < 0.001). Conversely, a significantly higher proportion of adolescents experienced intense pain compared to preschool and school-aged children (23.1% vs. 14.7%, *p* < 0.001). Differences in headache characteristics were observed between the two age groups. In preschool and school-aged children, the most common headache types, in order of frequency, were “other,” throbbing, pressure, and prickling pain. In contrast, adolescents most frequently experienced headaches in the following order: throbbing, pressure, and mixed pain. Notably, throbbing pain was more prevalent among adolescents (41.4% vs. 22.9%, *p* < 0.001), whereas there was no significant difference in the frequency of pressure-like pain between the two groups. The proportion of patients with headaches awakening from sleep (22.8% vs. 16.2%) and headaches upon waking in the morning (31.4% vs. 25.0%) was considerably higher in adolescents (*p* < 0.001).

Overall, adolescents reported headache-associated symptoms more frequently than preschool and school-aged children (74.1% vs. 60.4%, *p* < 0.001). Adolescents were more likely to experience a range of headache-associated symptoms, including nausea, vomiting, photophobia, phonophobia, and dizziness. In contrast, preschool and school-aged children more commonly experienced abdominal pain and nasal symptoms. A significantly higher proportion of adolescents had neurological abnormalities, including focal weakness, visual disturbances, hearing impairment, dysesthesia, decreased consciousness, and movement symptoms. Headache triggers were reported more frequently among adolescents than preschool and school-aged children (24.2% vs. 19.9%, *p* = 0.008), with emotional stress being the most commonly identified trigger in both groups. The proportion of adolescents who underwent neuroimaging was significantly higher than that of preschool and school-aged children (57.0% vs. 44.8%, *p* < 0.001). The proportion of patients receiving treatment for headaches, including analgesics, triptans, and prophylaxis, was significantly higher among adolescents (*p* < 0.001). No statistically significant between-group differences were observed regarding the presence of a family history of migraines.

### 3.3. Comparison of Secondary Headache Characteristics by Age Groups

Of the 707 patients, 545 (77.1%) were preschool and school-aged children, and 162 (22.9%) were adolescents. As shown in Table 4, the most common secondary diagnoses were headaches attributed to infection, accounting for 90.8% of preschool and school-aged children and 80.2% of adolescents. The prevalence of headaches attributed to infection was significantly higher in preschool and school-aged children (90.8% vs. 80.2%, *p* < 0.001), whereas adolescents exhibited significantly higher frequencies of cranial and cervical vascular disorders (3.7% vs. 1.3%, *p* = 0.044) and psychiatric disorders (5.6% vs. 0.6%, *p* < 0.001). Preschool and school-aged children more frequently experienced acute headaches with a combination of diffuse and localized pain; however, adolescents more frequently experienced recurrent and chronic headaches with localized or diffuse pain. Both groups experienced pain of moderate severity most frequently. However, the proportion of adolescents reporting severe pain was significantly higher than that of preschool and school-aged children (13.6% vs. 6.6%, *p* = 0.005). No significant differences were observed between the two groups in the duration and frequency of headaches, headaches upon awakening from sleep, or headaches upon waking in the morning. Preschool and school-aged children presented with headaches categorized as “other” more frequently compared to adolescents. Conversely, adolescents were more likely to experience headaches characterized by pressure (27.8% vs. 8.4%) or throbbing pain (14.8% vs. 6.1%) compared to preschool and school-aged children (*p* < 0.001).

Regarding headache-associated symptoms, both groups experienced nausea (58.9%) and vomiting (58.0%) frequently. However, a significantly higher proportion of adolescents presented with photophobia, phonophobia, and dizziness. The proportion of patients presenting with dizziness was significantly higher than that of preschool and school-aged children (24.1% vs. 7.7%, *p* < 0.001). Overall, adolescents reported neurological abnormalities more frequently than preschool- and school-aged children (11.7% vs. 2.4%, *p* < 0.001), with a significantly higher proportion of adolescents experiencing visual disturbances (8.0% vs. 0.9%, *p* < 0.001). Headache triggers were reported more frequently among adolescents than among preschool and school-aged children (8.0% vs. 2.0%, *p* < 0.001), with emotional stress and fatigue being the most commonly identified triggers in the adolescent group. The proportion of adolescents who underwent neuroimaging was significantly higher than that of preschool and school-aged children (34.6% vs. 18.9%, *p* < 0.001).

## 4. Discussion

This was a relatively large-scale study of the clinical characteristics of pediatric headaches based on the underlying etiology and age groups. In clinical practice, identifying pediatric patients with headaches who require extensive diagnostic investigations is challenging because of the difficulty in obtaining accurate information from children. In this regard, this study is noteworthy for demonstrating differences in patient distribution by age, headache characteristics, and the prevalence of headache-associated symptoms, neurological abnormalities, and headache triggers between the primary and secondary headache groups. Notably, preschool and school-aged children and adolescents exhibited differences in headache characteristics, distribution of headache-associated symptoms, neurological abnormalities, headache triggers, and etiology of secondary headaches.

Diagnosing primary headaches in children is challenging due to nonspecific symptoms and difficulty in articulating pain, which can delay diagnosis and hinder the identification of specific headache types [18]. A clear diagnostic distinction between migraines and tension-type headaches is not possible in 30.0–50.0% of children [19], which aligns with our discovery that 48.3% of primary headaches in preschool and school-aged children were unclassified. Preschool and school-aged children with primary headaches tended to experience acute and less recurrent headaches of mild severity. The duration of headache attacks in preschool and school-aged children was usually shorter than in adolescents, lasting less than 30 min. The higher likelihood of preschool children describing headaches as “other” and the lower likelihood of reporting associated symptoms, neurologic abnormalities, or triggers, may be attributed to their limited ability to accurately convey such information. Headaches in children can impair psychosocial functioning, affecting family relationships, leisure, work, and school productivity. They may also increase the risk of physical and mental health issues, such as anxiety and depression, in adulthood [20]. Considering that a longer interval between headache onset and initial medical consultation is associated with worse outcomes [2], ensuring early diagnosis and appropriate management for children with primary headaches is crucial. A comprehensive understanding of age-specific clinical characteristics can enhance this process.

Female adolescents had a higher proportion of primary headaches compared with male adolescents, whereas the proportions were similar among preschool and school-aged children. Consistent with our findings, Foiadelli et al. [21] conducted a study of 1950 adolescents and reported a headache prevalence of 65.9%, with females experiencing headaches more frequently than males at a ratio of 1.25:1. A cross-sectional study of 3423 children and adolescents found no significant difference in the one-year prevalence of primary headaches between males and females in the 6–11 years age group at 10.1% and 10.6%, respectively. In contrast, in the 12–17 years age group, the prevalence was significantly higher in females than in males (38.1% and 15.8%, respectively) [22]. The higher prevalence of primary headaches among females during adolescence may be related to puberty and the effects of estrogen on smooth muscles, particularly in the intracranial blood vessels [23].

Our data revealed that in the primary headache group, preschool and school-aged children tended to be underweight, whereas adolescents were often overweight. This contrast in weight trends suggests that headache risk factors may differ by age. Consistent with our findings, Robberstad et al. [24] conducted a study of 5847 adolescents and reported that the risk of migraine was 60% higher among those who were overweight or obese. The mechanisms underlying the association between migraines and body composition are not fully understood [25]. Obesity is characterized by systemic inflammation and increased proinflammatory cytokines [26,27]. Adipose tissue releases adipocytokines that promote this proinflammatory state [28]. Elevated levels of proinflammatory markers and adipocytokines have been particularly noted in patients with migraine [29,30]. Another potential mechanism involves the elevated plasma concentrations of calcitonin gene-related peptides in individuals with obesity, which influences meningeal nociception and pain transmission in the brain [31]. As obesity and being underweight are potentially modifiable risk factors for pediatric headaches, clinicians should be aware of these factors and promote healthy lifestyles, including proper diet, exercise, and weight management when treating pediatric patients with headaches.

In this study, the prevalence of primary headaches was considerably higher in preschool and school-aged children (59.9%) than in adolescents (40.1%). The current findings are consistent with those of previous studies that have demonstrated an increasing incidence of primary headaches among school-aged children in recent decades [32]. This phenomenon may be attributed to lifestyle changes, including increased screen time, altered sleep patterns, diet, stress, and greater awareness of headaches among parents and clinicians [18]. This study also demonstrated that the secondary headache group exhibited clinical characteristics that were distinct from those of the primary headache group. Patients with secondary headaches were generally younger and presented with acute headaches characterized by diffuse or localized pain of a mixed nature, often lasting for more than 1 h. The proportion of patients with severe headache was lower in the secondary headache group (58/707, 8.2%) than in the primary headache group (482/2667, 18.1%). Although the secondary headache group exhibited a lower rate of identifiable headache triggers than the primary headache group, the proportion of adolescents with headache triggers in the secondary headache group was particularly high (13/162, 8.0%). Our study found that emotional stress was the most common trigger for primary headaches in preschoolers, school-aged children, and adolescents. A consistent pattern of headache attacks following specific triggers may aid in determining the likelihood of primary headache [33]. Our efforts to delineate the clinical characteristics of patients with primary or secondary headaches could help identify those who require a comprehensive evaluation, thereby reducing unnecessary testing and improving the efficiency of diagnosis and treatment.

Clinicians usually look for red flags, including headaches upon awakening or sleep interruptions, to determine the need for neuroimaging, as these may indicate increased intracranial pressure [34]. However, a previous study reported that in clinically healthy and neurologically normal pediatric patients, headaches upon awakening or those that wake an individual from sleep are more commonly associated with primary headaches, particularly migraines or tension-type headaches. Other potential causes, such as sleep apnea, nocturnal hypertension, hypoglycemia, and medication-overuse headaches, should also be considered and ruled out [35]. Similarly, our study revealed that the proportion of patients experiencing headaches that awakened them from sleep or upon waking in the morning was relatively lower in the secondary headache group (17.5%) than in the primary headache group (46.5%). In addition, we found that the primary headache group had a significantly higher proportion of patients with neurological symptoms, including motor weakness, visual disturbances, changes in sensation, and movement symptoms. It is possible that the patients may have misinterpreted certain migraine features as the symptoms mentioned above, which may have contributed to these results. Therefore, clinical follow-ups are warranted for patients reporting transient or subjective symptoms. Conversely, this study noted a relatively higher prevalence of gait abnormalities, suggesting that patients with headaches accompanied by abnormalities on neurological examinations should be promptly investigated to identify the potential underlying causes.

Excluding primary headaches, most pediatric headaches have secondary causes, such as non-life-threatening infections [36]. In a study of 150 children with acute headaches, upper respiratory tract infections with fever were the leading cause, accounting for 57% of cases [37]. Similarly, our findings showed that infection-related headaches were the predominant cause of secondary headaches across all age groups, with upper respiratory tract infections (47%), acute gastroenteritis (27.2%), and central nervous system infections (19.8%). These headaches typically present with systemic symptoms such as fever, malaise, vomiting, lethargy, or neck stiffness [38]. Therefore, distinguishing these secondary headaches requires a thorough history taking and examination. Additionally, headaches attributable to psychiatric disorders were also commonly observed in adolescents in this study. This finding aligns with a population-based study demonstrating that symptoms of anxiety, depression, and behavioral problems were associated with recurrent headaches among Norwegian adolescents [39]. The pathophysiology of headaches related to stress, anxiety, and depression is multifactorial, involving mood dysregulation, sleep disturbances, and reduced physical activity [23]. Therefore, routine sleep and stress screening is recommended for adolescents with headaches. Increased awareness, along with the identification and treatment of comorbid psychiatric conditions, is crucial for improving the management and prognosis of adolescents with recurrent headaches. Patient education, particularly when initiated early at diagnosis and continued throughout treatment, plays a key role in improving headache management [40]. This multidisciplinary approach can enhance functional abilities and support regular school attendance [23].

This study had certain limitations. First, owing to the retrospective nature of the study, not all data were available, which may have resulted in underreporting if the data were missed in the chart review. Consequently, in some patients, the temporal relationship between headache, intracranial abnormalities, and headache response after treatment has not been fully investigated. This limitation may be compounded by the difficulty of obtaining accurate information from preschool children, which could have led to classifying patients with probable migraine or probable tension-type headaches as unclassified primary headaches, since these conditions were not distinctly categorized. Additionally, the retrospective design made it difficult to assess the role of medication overuse in some patients with chronic headaches, potentially leading to underdiagnosis of medication-overuse headaches. The lack of detailed data on headache onset, medication use, and the effects of discontinuation further complicated medication-overuse headache classification. Second, as the data were collected from two referral hospitals, the findings may not be representative of the general population. Selection bias favoring institutionalization is unavoidable. Thus, larger prospective studies or collaborative trials are warranted to further elucidate the clinical characteristics of pediatric headaches based on more detailed age groups and to identify the sociodemographic and socioeconomic factors that predict their occurrence.

## 5. Conclusions

Preschool and school-aged children and adolescents exhibited differences in headache characteristics, distribution of headache-associated symptoms, neurological abnormalities, headache triggers, and etiology of secondary headaches. Secondary headaches attributed to infections account for most headaches in preschool and school-aged children and adolescents. Headaches attributable to psychiatric disorders were commonly observed in adolescents. Therefore, we recommend recognizing age-specific differences in the clinical characteristics of headaches and tailoring diagnostic approaches accordingly. Furthermore, employing a multidisciplinary approach and addressing comorbid psychiatric conditions are essential for achieving optimal outcomes in pediatric patients with headaches.

## Figures and Tables

**Table 1:** Patient characteristics.

	Number of Patients (n = 3374)
Male, n (%)	1593 (47.2%)
Age (years) at initial visit, median (IQR)	11 (7–14)
Types of headaches	
Primary headaches (n = 2667, 79.0%)	
Migraine without aura	993 (37.2%)
Migraine with aura	147 (5.5%)
Tension-type headache	474 (17.8%)
Unclassified	1053 (39.5%)
Secondary headaches (n = 707, 21.0%)	
Cranial and cervical vascular disorder (n = 13, 1.8%)	
Moyamoya disease	5 (38.5%)
Arteriovenous malformation	2 (15.4%)
Cavernous malformation	2 (15.4%)
Cerebral aneurysm	1 (7.7%)
Internal carotid artery agenesis	1 (7.7%)
Reversible cerebral vasoconstriction syndrome	1 (7.7%)
MELAS	1 (7.7%)
Non-vascular intracranial disorder (n = 13, 1.8%)	
Brain tumor	10 (76.9%)
Chiari malformation	2 (15.4%)
Idiopathic intracranial hypertension	1 (7.7%)
Infection (n = 625, 88.4%)	
Upper respiratory tract infection	294 (47.0%)
Lower respiratory tract infection	3 (0.5%)
Acute gastroenteritis	170 (27.2%)
Urinary tract infection	2 (0.3%)
Central nervous system infection	124 (19.8%)
Fever without a focus	32 (5.1%)
Disorder of homeostasis (n = 5, 0.7%)	
Disorder of the cranium, neck, eyes, ears, nose, sinuses	
teeth, mouth, other facial or cervical structure (n = 39, 5.5%)	
Psychiatric disorder (n = 12, 1.7%)	

IQR, interquartile range; MELAS, mitochondrial encephalomyopathy with lactic acidosis and stroke-like episodes.

**Table 2:** Comparison of headache characteristics between the primary and secondary headache groups.

	Primary Headaches (n = 2667)	Secondary Headaches (n = 707)	*p* Value
Male, n (%)	1209 (45.3%)	384 (54.3%)	<0.001
Age (years) at initial visit, median (IQR)	11 (8–14)	8 (5–12)	
Age at initial visit			<0.001
Preschool age (≤6 years)	434 (16.3%)	265 (37.5%)	<0.001
Children (7–12 years)	1163 (43.6%)	280 (39.6%)	0.056
Adolescent (13–18 years)	1070 (40.1%)	162 (22.9%)	<0.001
Clinical course			<0.001
Acute (≤3 months)	816 (30.6%)	659 (93.2%)	
Acute recurrent (≤3 months)	530 (19.9%)	22 (3.1%)	
Chronic (>3 months)	375 (14.1%)	7 (1.0%)	
Chronic progressive (>3 months)	943 (35.4%)	19 (2.7%)	
Mixed	3 (0.1%)	0	
Localization			<0.001
Diffuse	422 (15.8%)	78 (11.0%)	
Localized	2010 (75.4%)	273 (38.6%)	
Mixed	235 (8.8%)	356 (50.4%)	
Duration			<0.001
<10 min	320 (12.0%)	25 (3.5%)	
10–<30 min	308 (11.5%)	24 (3.4%)	
30–<60 min	376 (14.1%)	29 (4.1%)	
≥1 h	1663 (62.4%)	629 (89.0%)	
Frequency			0.015
<2/month	261 (9.8%)	56 (7.9%)	0.131
2–<4/month	468 (17.5%)	117 (16.5%)	0.533
4–<15/month	869 (32.6%)	231 (32.7%)	0.964
≥15/month	807 (30.3%)	253 (35.8%)	0.005
Daily	262 (9.8%)	50 (7.1%)	0.025
Pain intensity			<0.001
Mild	547 (20.5%)	72 (10.2%)	<0.001
Moderate	1638 (61.4%)	577 (81.6%)	<0.001
Severe	482 (18.1%)	58 (8.2%)	<0.001
Headache awakening from sleep at night	503 (18.9%)	66 (9.3%)	<0.001
Headache on or soon after waking up in the morning	736 (27.6%)	58 (8.2%)	<0.001
Character			<0.001
Throbbing	808 (30.3%)	57 (8.1%)	<0.001
Sharp	112 (4.2%)	18 (2.5%)	0.042
Cramping	27 (1.0%)	4 (0.6%)	0.375
Prickling	254 (9.5%)	30 (4.2%)	<0.001
Constant/dull	133 (5.0%)	25 (3.5%)	0.105
Pressure	626 (23.5%)	91 (12.9%)	<0.001
Mixed	189 (7.1%)	473 (66.9%)	<0.001
Others	518 (19.4%)	9 (1.3%)	<0.001
Associated symptoms	1757 (65.9%)	523 (74.0%)	<0.001
Nausea/vomiting	1167 (43.8%)	415 (58.7%)	<0.001
Abdominal pain	172 (6.4%)	85 (12.0%)	<0.001
Photophobia	398 (14.9%)	17 (2.4%)	<0.001
Phonophobia	432 (16.2%)	23 (3.3%)	<0.001
Dizziness	804 (30.1%)	81 (11.5%)	<0.001
Nasal symptoms	106 (4.0%)	98 (13.9%)	<0.001
Presence of neurologic abnormalities *	415 (15.6%)	32 (4.5%)	<0.001
Gait disturbance	4 (0.1%)	2 (0.3%)	0.612
Focal weakness	55 (2.1%)	6 (0.8%)	0.031
Visual disturbance	295 (11.1%)	18 (2.5%)	<0.001
Hearing impairment	19 (0.7%)	1 (0.1%)	0.098
Dysarthria/aphasia	11 (0.4%)	2 (0.3%)	1.00
Dysesthesia	51 (1.9%)	4 (0.6%)	0.011
Decreased consciousness	27 (1.0%)	5 (0.7%)	0.662
Seizure	6 (0.2%)	1 (0.1%)	1.00
Movement symptom ^†^	25 (0.9%)	0	0.005
Triggers	577 (21.6%)	24 (3.4%)	<0.001
Emotional stress	376 (14.1%)	13 (1.8%)	<0.001
Hunger	12 (0.4%)	2 (0.3%)	0.747
Weather	89 (3.3%)	6 (0.8%)	<0.001
Fatigue	131 (4.9%)	8 (1.1%)	<0.001
Exercise	74 (2.8%)	2 (0.3%)	<0.001
Light	22 (0.8%)	1 (0.1%)	0.067
Noise	23 (0.9%)	1 (0.1%)	0.043
Smell	31 (1.2%)	0	0.001
Neuroimaging			
Performed	1325 (49.7%)	159 (22.5%)	<0.001
Computed tomography	94 (7.1%)	54 (34.0%)	
Magnetic resonance imaging	1231 (92.9%)	105 (66.0%)	
Family history of migraine	831 (31.2%)	32 (4.5%)	<0.001

IQR, interquartile range. * Within 6 months prior to visit. ^†^ Tremor, myoclonus.

**Table 3:** Comparison of headache characteristics among different age groups of patients with primary headaches.

	Preschool, School-Aged Children (n = 1597)	Adolescents (n = 1070)	*p* Value
Male, n (%)	812 (50.8%)	397 (37.1%)	<0.001
Age (years) at initial visit, median (IQR)	9 (6–11)	15 (14–16)	
Body mass index			<0.001
<18.5	608 (38.1%)	166 (15.5%)	<0.001
18.5–<24.9	846 (53.0%)	696 (65.0%)	<0.001
≥25	143 (9.0%)	208 (19.4%)	<0.001
Types of headaches			<0.001
Migraine without aura	480 (30.1%)	513 (47.9%)	<0.001
Migraine with aura	51 (3.2%)	96 (9.0%)	<0.001
Tension-type headache	294 (18.4%)	180 (16.8%)	0.293
Unclassified	772 (48.3%)	281 (26.3%)	<0.001
Clinical course			<0.001
Acute (≤3 months)	551 (34.5%)	265 (24.8%)	<0.001
Acute recurrent (≤3 months)	339 (21.2%)	191 (17.9%)	0.032
Chronic (>3 months)	193 (12.1%)	182 (17.0%)	<0.001
Chronic progressive (>3 months)	513 (32.1%)	430 (40.2%)	<0.001
Mixed	1 (0.1%)	2 (0.2%)	0.568
Localization			<0.001
Diffuse	226 (14.2%)	196 (18.3%)	0.004
Localized	1201 (75.2%)	809 (75.6%)	0.812
Mixed	170 (10.6%)	65 (6.1%)	<0.001
Duration			<0.001
<10 min	247 (15.5%)	73 (6.8%)	<0.001
10–<30 min	213 (13.3%)	95 (8.9%)	<0.001
30–<60 min	238 (14.9%)	138 (12.9%)	0.145
≥1 h	899 (56.3%)	764 (71.4%)	<0.001
Frequency			0.036
<2/month	285 (17.8%)	227 (21.2%)	0.030
2–<4/month	289 (18.1%)	223 (20.8%)	0.078
4–<15/month	434 (27.2%)	265 (24.8%)	0.165
≥15/month	424 (26.5%)	253 (23.6%)	0.091
Daily	165 (10.3%)	102 (9.5%)	0.500
Pain intensity			<0.001
Mild	415 (26.0%)	132 (12.3%)	<0.001
Moderate	947 (59.3%)	691 (64.6%)	0.006
Severe	235 (14.7%)	247 (23.1%)	<0.001
Character			<0.001
Throbbing	365 (22.9%)	443 (41.4%)	<0.001
Sharp	76 (4.8%)	36 (3.4%)	0.078
Cramping	18 (1.1%)	9 (0.8%)	0.470
Prickling	193 (12.1%)	61 (5.7%)	<0.001
Constant/dull	92 (5.8%)	41 (3.8%)	0.025
Pressure	359 (22.5%)	267 (25.0%)	0.140
Mixed	74 (4.6%)	115 (10.7%)	<0.001
Others	420 (26.3%)	98 (9.2%)	<0.001
Headache awakening from sleep at night	259 (16.2%)	244 (22.8%)	<0.001
Headache on or soon after waking up in the morning	400 (25.0%)	336 (31.4%)	<0.001
Associated symptoms	964 (60.4%)	793 (74.1%)	<0.001
Nausea/vomiting	604 (37.8%)	563 (52.6%)	<0.001
Abdominal pain	116 (7.3%)	56 (5.2%)	0.036
Photophobia	191 (12.0%)	207 (19.3%)	<0.001
Phonophobia	180 (11.3%)	252 (23.6%)	<0.001
Dizziness	432 (27.1%)	373 (34.9%)	<0.001
Nasal symptoms	81 (5.1%)	25 (2.3%)	<0.001
Presence of neurologic abnormalities *	163 (10.2%)	252 (23.6%)	<0.001
Gait disturbance	3 (0.2%)	1 (0.1%)	0.653
Focal weakness	21 (1.3%)	34 (3.2%)	<0.001
Visual disturbance	117 (7.3%)	178 (16.6%)	<0.001
Hearing impairment	7 (0.4%)	12 (1.1%)	0.040
Dysarthria/aphasia	6 (0.4%)	5 (0.5%)	0.718
Dysesthesia	23 (1.4%)	28 (2.6%)	0.030
Decreased consciousness	7 (0.4%)	20 (1.9%)	<0.001
Seizure	2 (0.1%)	4 (0.4%)	0.227
Movement symptom ^†^	5 (0.3%)	20 (1.9%)	<0.001
Triggers	318 (19.9%)	259 (24.2%)	0.008
Emotional stress	195 (12.2%)	181 (16.9%)	<0.001
Hunger	8 (0.5%)	4 (0.4%)	0.772
Weather	56 (3.5%)	33 (3.1%)	0.552
Fatigue	74 (4.6%)	57 (5.3%)	0.417
Exercise	43 (2.7%)	31 (2.9%)	0.752
Light	15 (0.9%)	7 (0.7%)	0.425
Noise	8 (0.5%)	15 (1.4%)	0.014
Smell	14 (0.9%)	17 (1.6%)	0.093
Treatment			
Analgesics (acetaminophen, ibuprofen)	1151 (72.1%)	977 (91.3%)	<0.001
Triptans	2 (0.1%)	32 (3.0%)	<0.001
Prophylaxis	61 (3.8%)	166 (15.5%)	<0.001
Flunarizine	18 (29.5%)	102 (61.4%)	
Amitriptyline	23 (37.7%)	50 (30.1%)	
Propranolol	4 (6.6%)	33 (19.9%)	
Topiramate	25 (41.0%)	39 (23.5%)	
Others	1 (1.6%)	11 (6.6%)	
Neuroimaging			
Performed	715 (44.8%)	610 (57.0%)	<0.001
Computed tomography	32 (2.0%)	62 (10.2%)	
Magnetic resonance imaging	683 (42.8%)	548 (89.8%)	
Family history of migraine	513 (32.1%)	318 (29.7%)	0.189

IQR, interquartile range. * Within 6 months prior to visit. ^†^ Tremor, myoclonus.

**Table 4:** Comparison of headache characteristics among different age group of patients with secondary headaches.

	Preschool, School-Aged Children (n = 545)	Adolescents (n = 162)	*p* Value
Male, n (%)	302 (55.4%)	82 (50.6%)	0.282
Age (years) at initial visit, median (IQR)	7 (5–10)	15 (13–16)	
Types of headaches			<0.001
Cranial and cervical vascular disorder	7 (1.3%)	6 (3.7%)	0.044
Non-vascular intracranial disorder	8 (1.5%)	5 (3.1%)	0.178
Infection	495 (90.8%)	130 (80.2%)	<0.001
Disorder of homeostasis	2 (0.4%)	3 (1.9%)	0.082
Disorder of the cranium, neck, eyes, ears,	30 (5.5%)	9 (5.6%)	0.980
nose, sinuses, teeth, mouth, other facial or			
cervical structure			
Psychiatric disorder	3 (0.6%)	9 (5.6%)	<0.001
Clinical course			<0.001
Acute (≤3 months)	518 (95.0%)	141 (87.0%)	<0.001
Acute recurrent (≤3 months)	16 (2.9%)	6 (3.7%)	0.621
Chronic (>3 months)	3 (0.6%)	4 (2.5%)	0.052
Chronic progressive (>3 months)	8 (1.5%)	11 (6.8%)	<0.001
Localization			<0.001
Diffuse	49 (9.0%)	29 (17.9%)	0.001
Localized	191 (35.0%)	82 (50.6%)	<0.001
Mixed	305 (56.0%)	51 (31.5%)	<0.001
Duration			0.219
<10 min	17 (3.1%)	8 (4.9%)	
10–<30 min	15 (2.8%)	9 (5.6%)	
30–<60 min	22 (4.0%)	7 (4.3%)	
≥1 h	491 (90.1%)	138 (85.2%)	
Frequency			0.580
<2/month	72 (13.2%)	27 (16.7%)	
2–<4/month	71 (13.0%)	19 (11.7%)	
4–<15/month	188 (34.5%)	52 (32.1%)	
≥15/month	166 (30.5%)	45 (27.8%)	
Daily	48 (8.8%)	19 (11.7%)	
Pain intensity			0.016
Mild	58 (10.6%)	14 (8.6%)	0.460
Moderate	451 (82.8%)	126 (77.8%)	0.151
Severe	36 (6.6%)	22 (13.6%)	0.005
Character			<0.001
Throbbing	33 (6.1%)	24 (14.8%)	<0.001
Sharp	14 (2.6%)	4 (2.5%)	1.00
Cramping	4 (0.7%)	0	0.579
Prickling	20 (3.7%)	10 (6.2%)	0.165
Constant/dull	15 (2.8%)	10 (6.2%)	0.038
Pressure	46 (8.4%)	45 (27.8%)	<0.001
Mixed	5 (0.9%)	4 (2.5%)	0.128
Others	408 (74.9%)	65 (40.1%)	<0.001
Headache awakening from sleep at night	47 (8.6%)	19 (11.7%)	0.233
Headache on or soon after waking up in the morning	39 (7.2%)	19 (11.7%)	0.063
Associated symptoms	399 (73.2%)	124 (76.5%)	0.396
Nausea/vomiting	321 (58.9%)	94 (58.0%)	0.843
Abdominal pain	69 (12.7%)	16 (9.9%)	0.339
Photophobia	9 (1.7%)	8 (4.9%)	0.016
Phonophobia	13 (2.4%)	10 (6.2%)	0.017
Dizziness	42 (7.7%)	39 (24.1%)	<0.001
Nasal symptoms	77 (14.1%)	21 (13.0%)	0.706
Presence of neurologic abnormalities *	13 (2.4%)	19 (11.7%)	<0.001
Gait disturbance	2 (0.4%)	0	1.00
Focal weakness	3 (0.6%)	3 (1.9%)	0.137
Visual disturbance	5 (0.9%)	13 (8.0%)	<0.001
Hearing impairment	0	1 (0.6%)	0.229
Dysarthria/aphasia	0	2 (1.2%)	0.052
Dysesthesia	2 (0.4%)	2 (1.2%)	0.227
Decreased consciousness	2 (0.4%)	3 (1.9%)	0.082
Seizure	1 (0.2%)	0	1.00
Movement symptom ^†^	0	0	
Triggers	11 (2.0%)	13 (8.0%)	<0.001
Emotional stress	5 (0.9%)	8 (4.9%)	<0.001
Hunger	1 (0.2%)	1 (0.6%)	0.406
Weather	3 (0.6%)	3 (1.9%)	0.137
Fatigue	3 (0.6%)	5 (3.1%)	0.018
Exercise	0	2 (1.2%)	0.052
Light	0	1 (0.6%)	0.229
Noise	1 (0.2%)	0	1.00
Smell	0	0	
Neuroimaging			
Performed	103 (18.9%)	56 (34.6%)	<0.001
Computed tomography	39 (7.2%)	15 (26.8%)	
Magnetic resonance imaging	64 (11.7%)	41 (73.2%)	
Family history of migraine	20 (3.7%)	12 (7.4%)	0.045

IQR, interquartile range. * Within 6 months prior to visit. ^†^ Tremor, myoclonus.

## Data Availability

The original contributions presented in the study are included in the article, further inquiries can be directed to the corresponding author.

## References

[1] GBD 2019 Diseases and Injuries Collaborators (2020). Global burden of 369 diseases and injuries in 204 countries and territories, 1990-2019: A systematic analysis for the Global Burden of Disease Study 2019. Lancet.

[2] Antonaci F., Voiticovschi-Iosob C., Di Stefano A.L., Galli F., Ozge A., Balottin U. (2014). The evolution of headache from childhood to adulthood: A review of the literature. J. Headache Pain.

[3] Gassmann J., Vath N., van Gessel H., Kröner-Herwig B. (2009). Risk factors for headache in children. Dtsch. Arztebl. Int..

[4] Anttila P., Metsähonkala L., Sillanpää M. (2006). Long-term trends in the incidence of headache in Finnish schoolchildren. Pediatrics.

[5] Jacobsena B.A., Dyb G., Hagen K., Stovner L.J., Holmen T.L., Zwart J.A. (2018). The Nord-Trøndelag Health Study shows increased prevalence of primary recurrent headaches among adolescents over a four-year period. Scand. J. Pain.

[6] Roser T., Bonfert M., Ebinger F., Blankenburg M., Ertl-Wagner B., Heinen F. (2013). Primary versus secondary headache in children: A frequent diagnostic challenge in clinical routine. Neuropediatrics.

[7] Burton L.J., Quinn B., Pratt-Cheney J.L., Pourani M. (1997). Headache etiology in a pediatric emergency department. Pediatr. Emerg. Care.

[8] Conicella E., Raucci U., Vanacore N., Vigevano F., Reale A., Pirozzi N., Valeriani M. (2008). The child with headache in a pediatric emergency department. Headache.

[9] Scagni P., Pagliero R. (2008). Headache in an Italian pediatric emergency department. J. Headache Pain.

[10] GBD 2016 Neurology Collaborators (2019). Global, regional, and national burden of neurological disorders, 1990–2016: A systematic analysis for the Global Burden of Disease Study 2016. Lancet Neurol..

[11] Arruda M.A., Bigal M.E. (2012). Behavioral and emotional symptoms and primary headaches in children: A population-based study. Cephalalgia.

[12] Onofri A., Olivieri L., Silva P., Bernassola M., Tozzi E. (2022). Correlation between primary headaches and learning disabilities in children and adolescents. Minerva Pediatr..

[13] Falla K., Kuziek J., Mahnaz S.R., Noel M., Ronksley P.E., Orr S.L. (2022). Anxiety and depressive symptoms and disorders in children and adolescents with migraine: A systematic review and meta-analysis. JAMA Pediatr..

[14] Powers S.W., Coffey C.S., Chamberlin L.A., Ecklund D.J., Klingner E.A., Yankey J.W., Peugh J.L., Korbee L.L., Simmons K., Sullivan S.M. (2021). Prevalence of headache days and disability 3 years after participation in the childhood and adolescent migraine prevention medication trial. JAMA Netw. Open.

[15] Singhi S., Jacobs H., Gladstein J. (2014). Pediatric headache: Where have we been and where do we need to be. Headache.

[16] Babineau S.E., Green M.W. (2012). Headaches in children. Continuum.

[17] Headache Classification Committee of the International Headache Society (IHS) (2018). The International Classification of Headache Disorders, 3rd edition. Cephalalgia.

[18] Abu-Arafeh I., Howells R. (2014). Primary headaches in children under the age of 7 years. Curr. Pain Headache Rep..

[19] Wager J., Hirschfeld G., Zernikow B. (2013). Tension-type headache or migraine? Adolescents’ pain descriptions are of little help. Headache.

[20] Guidetti V., Galli F., Fabrizi P., Giannantoni A.S., Napoli L., Bruni O., Trillo S. (1998). Headache and psychiatric comorbidity: Clinical aspects and outcome in an 8-year follow-up study. Cephalalgia.

[21] Foiadelli T., Piccorossi A., Sacchi L., De Amici M., Tucci M., Brambilla I., Marseglia G.L., Savasta S., Verrotti A. (2018). Clinical characteristics of headache in Italian adolescents aged 11-16 years: A cross-sectional questionnaire school-based study. Ital. J. Pediatr..

[22] Al-Hashel J.Y., Ahmed S.F., Alroughani R. (2019). Prevalence and Burden of Primary Headache Disorders in Kuwaiti Children and Adolescents: A Community Based Study. Front. Neurol..

[23] Langdon R., DiSabella M.T. (2017). Pediatric Headache: An Overview. Curr. Probl. Pediatr. Adolesc. Health Care.

[24] Robberstad L., Dyb G., Hagen K., Stovner L.J., Holmen T.L., Zwart J.A. (2010). An unfavorable lifestyle and recurrent headaches among adolescents: The HUNT study. Neurology.

[25] Martami F., Jayedi A., Shab-Bidar S. (2022). Primary headache disorders and body mass index categories: A systematic review and dose-response meta-analysis. Headache.

[26] Cancello R., Clément K. (2006). Is obesity an inflammatory illness? Role of low-grade inflammation and macrophage infiltration in human white adipose tissue. BJOG.

[27] Schmidt F.M., Weschenfelder J., Sander C., Minkwitz J., Thormann J., Chittka T., Mergl R., Kirkby K.C., Faßhauer M., Stumvoll M. (2015). Inflammatory cytokines in general and central obesity and modulating effects of physical activity. PLoS ONE.

[28] Cao H. (2014). Adipocytokines in obesity and metabolic disease. J. Endocrinol..

[29] Conti P., D’Ovidio C., Conti C., Gallenga C.E., Lauritano D., Caraffa A., Kritas S.K., Ronconi G. (2019). Progression in migraine: Role of mast cells and pro-inflammatory and anti-inflammatory cytokines. Eur. J. Pharmacol..

[30] Domínguez C., Vieites-Prado A., Pérez-Mato M., Sobrino T., Rodríguez-Osorio X., López A., Campos F., Martínez F., Castillo J., Leira R. (2018). Role of adipocytokines in the pathophysiology of migraine: A cross-sectional study. Cephalalgia.

[31] Recober A., Goadsby P.J. (2010). Calcitonin gene-related peptide: A molecular link between obesity and migraine?. Drug News Perspect..

[32] Abu-Arafeh I., Razak S., Sivaraman B., Graham C. (2010). Prevalence of headache and migraine in children and adolescents: A systematic review of population-based studies. Dev. Med. Child Neurol..

[33] Orr S.L. (2024). Headache in Children and Adolescents. Continuum.

[34] Park E.G., Yoo I.H. (2022). The diagnostic values of red flags in pediatric patients with headache. Brain Dev..

[35] Ahmed M.A.S., Ramseyer-Bache E., Taylor K. (2018). Yield of brain imaging among neurologically normal children with headache on wakening or headache waking the patient from sleep. Eur. J. Paediatr. Neurol..

[36] Walter S.M., Laderman M., Polk P. (2021). Pediatric Headache Attributed to Infection. Semin. Pediatr. Neurol..

[37] Lewis D.W., Qureshi F. (2000). Acute headache in children and adolescents presenting to the emergency department. Headache.

[38] Abend N.S., Younkin D., Lewis D.W. (2010). Secondary headaches in children and adolescents. Semin. Pediatr. Neurol..

[39] Blaauw B.A., Dyb G., Hagen K., Holmen T.L., Linde M., Wentzel-Larsen T., Zwart J.A. (2014). Anxiety, depression and behavioral problems among adolescents with recurrent headache: The Young-HUNT study. J. Headache Pain.

[40] Ali M.W., Musami U.B., Sa’ad F.K., Omoaghe C., Danimoh M.A., Ayoola Y.A., Kanu A.O. (2020). Profile of migraine patients in a developing country: A multicentre study. SN Compr. Clin. Med..

